# Preservation techniques of stem cells extracellular vesicles: a gate for manufacturing of clinical grade therapeutic extracellular vesicles and long-term clinical trials

**DOI:** 10.1080/23144599.2019.1704992

**Published:** 2020-01-20

**Authors:** Mohamed M. Bahr, Mohamed S. Amer, Khaled Abo-El-Sooud, Ahmed N. Abdallah, Omar S. El-Tookhy

**Affiliations:** a Surgery Department, Faculty of Veterinary Medicine, Cairo University, Giza, Egypt; b Pharmacology Department, Faculty of Veterinary Medicine, Cairo University, Giza, Egypt; c Pathology Department, Animal Health Research Institute, Cairo, Egypt

**Keywords:** Long-term preservation, stem cell, therapeutic extracellular vesicles, clinical trials

## Abstract

Extracellular vesicles (EVs) are nanosized vesicles released by different cells and have been separated from most of the body fluids. These vesicles play a central role in cell-to-cell communications as carry a distinct cargo including proteins, RNA species, DNAs, and lipids that are meant to be shipped and exchanged between cells at both systemic and paracrine levels. They serve in regulating normal physiological processes. EVs released from stem cells exert similar therapeutic effect to their originating cells. Clinical application of EVs requires the preparation of sufficient and viable active therapeutic EVs as well as implementing suitable methods for long-term preservation to expedite both their clinical and commercial uses. Cryopreservation is the most common method used to preserve decomposable biomaterials. However, cryopreservation causes cryoinjury to cells which therefore necessitate the use of cryoprotectants. Two types of cryoprotectants exist: penetrating and non-penetrating. In freeze drying, the watery content is sublimed from the product after it is frozen. This drying process is pertinent to thermo-liable substances and those unstable in aqueous solutions for prolonged storage periods. In spray drying technique, the solution containing EVs is firstly atomized, then droplets are rapidly converted into a dry powder using heated gas. Even with the exposure to high temperatures of the drying gas, spray drying is considered suitable for heat-sensitive materials. EVs are considered a promising cell-free therapy, but the lack of proper preservation limits its benefits. Preservation of EVs will initiate a vast amount of clinical trials on different species and different clinical problems.

## 1. Introduction

Multicellular organisms consist of more than one cell and cell-to-cell communication is a pivotal process for them to get the harmony of cells action, interaction and to regulate the multiple functions of different body parts of the living organisms. The information is commonly delivered from cell to another by direct interaction or secretion of soluble factors. It was recently discovered that most eukaryotic cells release membranous vesicle which affects both neighbouring and faraway cells [1]. Under the electron microscope, and for many years, these extracellular vesicles (EV) were considered as an artefact. EVs were first described about 30 years ago when multivesicular forms were observed in the extracellular space of reticulocytes discharge [2]. From that time on, EVs have been isolated from stem cells [3], cells of the immune and nervous systems [4] and many cancer cell lines [5]. EVs are not only in mammals but have been also identified in lower eukaryotic and prokaryotic cells [6]. Although EVs were considered as membrane debris that have no biological significance, the ability of EVs to stimulate adaptive immune responses, raise their importance in cell-to-cell interaction [7]. EVs isolated from most bodily fluids so, EVs not only have a role in the regulation of normal physiological processes, as tissue repair [8] blood coagulation [9], immune surveillance [7] and stem cell maintenance [3] but also in several pathological diseases such as tumorigenesis [10], spread pathogenic agents such as HIV-1 [11], and pathogenic cell-surface protein PrP^C^ [12]. New studies revealed that EVs could be used directly as materials for injured tissue regeneration and immune reaction alteration as it was used to motivate tissue repair in case of myocardial infarction [13] and cutaneous wound healing [14,15].

## 2. Types of extracellular vesicles

EVs are classified according to their cellular origin into three types: (a) exosomes, (b) microvesicles and (c) apoptotic bodies. Till now, there is no demarcation line between microvesicles and exosomes due to the inconsistencies in EVs purification protocols and the lack of complete vesicles characterization. Yet, these vesicles can be readily separated from each other by differential ultracentrifugation [16]. The large-sized class is microvesicles which are generated by budding from the plasma membrane, size range is 50:1500 nm and is sedimented by centrifugation at 10,000:14,000× *g*. The small-sized class is exosomes which are derived from the endolysosomal pathway, size range is 50:120 nm and is sedimented by centrifugation at 100,000× *g* [17]. Apoptotic bodies are discharged by cells undergoing apoptosis and its size range is 50:2000 nm.

## 3. Biological actions of extracellular vesicles on the cells

As EVs are released by different cell types, they undoubtedly carry diverse cargoes. These cargos including proteins, RNA species (as mRNA, miRNA, lncRNA, and other RNA species), DNAs (mtDNA, ssDNA, dsDNA), as well as lipids that can be transported and exchanged between cells at both systemic and paracrine levels [18,19]. EVs exert their effect through different routes (a) activate cell surface receptor by proteins and bioactive lipids, (b) merging their membrane with the recipient cell plasma membrane and releasing their specific cargoes into recipient cells such as transcription factors, oncogenes, small and large non-coding regulatory RNAs, mRNAs and infectious particles [1,20]. EVs are able to regulate immune responses, depending on the status of particular immune cells, EVs might trigger adaptive immune responses or suppress inflammation [21]. EVs have been involved in cell phenotype modulation, as in case of converting the haematopoietic stem cell phenotype into a liver cell phenotype [22]. Several reports have shown that stem cell-derived EVs have a pivotal role in tissue regeneration following injury [10,13,23].

## 4. Therapeutic effect of EVs

EV-based therapeutics is based on the recently discovered fact that EVs shed by stem cells exert similar therapeutic effects to their originating cells. Mesenchymal stem cell (MSC)-based therapy relied on the idea that engrafted cells within damaged tissues will differentiate to replace injured cells. However, in myocardial infarction treated by transplanted MSCs, MSCs concentrated in other tissues than the myocardium, and showed slow and inefficient transdifferentiation into cardiomyocytes, however, the cardiac function returned more rapidly [24]. These findings led to the hypothesis that soluble factors released by MSCs are responsible for the beneficial outcomes which was called the paracrine effect. The paracrine effect depends on the secreted EVs, particularly exosomes [15,25]. EVs derived from stem cell have the ability to induce angiogenesis in endothelial cells [26], inhibition to cell apoptosis and stimulate proliferation of the cells [27], deliver immunomodulatory signals [7] and reprogramming the cells which are required for tissue regeneration [3]. Due to therapeutic effect of EVs that make it helpful in clinical application for treatment, manufacturing of clinical grade EVs to meet market demands must be have suitable formulation and preserved by suitable technique.

## 5. Clinical application of EV-based therapeutics

The regenerative effect of progenitor cells and stem cells can be obtained by EVs of each type of cells. This effect can play an important role in several diseases treatment. So many researches used EVs which derived from progenitor cells and stem cells as therapeutic agents (Table 1).

**Table 1.** Individual human-derived EVs and their therapeutic effects

EV source	Therapeutic effect	Reference
hMSCs	Increased engineered cardiac tissue	[28]
UCB plasma	Enhanced angiogenesis and promoted wound healing	[29]
hMSCs	Suppression of angiogenesis and	[30]
hMSCs	M2 polarization and increased survival	[31]
CMPCs and MSCs	Increased angiogenesis and endothelial cell migration	[32]
hBMMSCs and UC-MSCs	Increased cell recovery following injury	[33]
hMSCs	Reduced renal fibrosis	[34]
hBMMSCs	Ameliorated osteopenia	[35]
hMSCs	Restored cardiac contractile function and reduced infarct size	[36]
iPSCs	Rescued ischaemic cardiomyocytes	[37]

hMSCs: human mesenchymal stem cells; UCB: umbilical cord blood; CMPCs: cardiomyocyte progenitor cells; hBMMSCs: human bone marrow MSCs; UC-MSCs: umbilical cord MSCs; iPSCs: induced pluripotent stem cells.

## 6. Preservation strategies of extracellular vesicles

The aim of EVs preservation is to reach high viability percentage after the preservation process to save the EVs effectiveness and to obtain a medical form that can be easily transported and handled. There are two main techniques for the preservation of EVs, by deep freezing and by drying.

### 6.1. Cryopreservation

Cryopreservation is considered as a successful method to preserve cells function by lowering temperature below the required temperature for biochemical reaction, unfortunately cell cryopreservation is usually associated with “cryoinjury” [38]. This cryoinjury is due to (i) osmotic imbalance within freezing procedure (solution effects injury) and (ii) intracellular ice formation. In cell suspension, when ice forms during cooling, the ice crystals include only water molecules. As a result, all other components (salts, etc.) will be concentrated in the remaining solution. So, the concentration of the solution increases. When cell suspension freezes, the cells are trapped in channels of concentrated unfrozen medium. An osmotic gradient was generated through the cell membrane by the high concentration of this unfrozen solution resulting in water flow out of the cell through exosmosis. Exosmosis takes place when the solution slowly cooled, the water moves from intracellular to the outside of the cell to a sufficient degree to maintain near-equilibrium state of the cells with the extracellular liquid, where the ice will not form intracellularly. On the other hand, rapid cooling is resulting in escape of water slowly that not enough to preserve cell near-equilibrium state with the solution extracellularly, as a result of the formation of ice intracellularly which eventually resulting in cell death [39].

#### 6.1.1. Challenges associated with EVs cryo-preservation

In order to have viable and effective EVs, the preservation technique should be protect the EVs contents during both preservation and preparation for usage after preservation. The cryopreservation includes freezing of the EVs, thawing and possible re-freezing after partial usage. These steps could be harmful to the EVs. To overcome the dangers associated with freezing, cryopreservation is commonly associated with the addition of one or more compounds to protect cells during freezing, those compounds are called “cryoprotectants” which are characterized by simplicity, high water solubility and low toxicity [38].

Cryoprotectants are divided into two main classes: (a) intracellular agents (penetrating cryoprotectants), which penetrate into the cells to prevent ice crystals formation and subsequently membrane rupture (e.g. dimethyl sulphoxide (CH_3_)_2_SO, glycerol, and ethylene glycol) and (b) extracellular compounds (non-penetrating cryoprotectant), which does not penetrate cell membrane such as sucrose, trehalose and other sugars [40].

The two classes are different in mechanism of action depending on their molecular weight. Penetrating cryoprotectants work by infusing through the lipid bilayer membranes to stabilize the biomolecule as they have low molecular weights (<100 Da). On contrary, non-penetrating cryoprotectant becomes outside to the vesicle as a result of their high molecular mass (180–594 Da) that avoid cryoinjury from hyperosmotic lysis [41]. Many evidences suggested combining of both penetrating cryoprotectants and non-penetrating cryoprotectants to be more effective [42]. It’s noteworthy that the concentration of cryoprotectant should be adequately adjusted, because excessively low concentrations of cryoprotectants may lead to chilling shock (the damage caused by the freezing process), while, excessively high concentrations of cryoprotectants can be toxic. Thus, a maintained balance is required to achieve optimal cryopreservation [43].

#### 6.1.2. Penetrating cryoprotectants

Penetrating cryoprotectants can move across cell membranes and prepare the environment for reduction of cell water content [44]. This is performed at suitable low temperature enough to minimize the harmful effect of the concentrated solutes on the cells. The penetrating cryoprotectant also lead to membrane lipid and protein rearrangement, which results in increased membrane permeability, better dehydration at lower temperatures and improved cell ability to endure the cryopreservation processes [45].

(i) Glycerol is a penetrating cryoprotectant that forms hydrogen bonds with water molecules and renders a mixture of (70% glycerol and 30% water) difficult to form ice-crystals until the temperature is as low as (−37.8°C). Compared to other cryoprotectants, glycerol is less toxic in high concentration [46], but has a weakness due to its slow movement across permeable membranes [47].

(ii) Dimethyl sulphoxide (DMSO) is an organosulfur compound and it freezes at 18.5°C. This means, below room temperature, the DMSO becomes solid, and this physical character makes it most suitable as a cryoprotectant [48].

(iii) Ethylene glycol changes the hydrogen bonding when mixes with water. The freezing point of purified ethylene glycol is about −12°C, but after mixing with 40% water and 60% ethylene glycol the freezing point of the mixture would drop and the mixture becomes unable to form crystalline substances. This condition leads to lower the freezing point to −45°C. This property of ethylene glycol makes it the most effective cryoprotectant agent [49].

#### 6.1.3. Non-penetrating cryoprotectants

Non-penetrating cryoprotectants are composed of polymers which can form extensive hydrogen bonds with water and can’t move across cell membranes. At the primary phases, they osmotically “press” water out of the cells at temperatures between −10 and −20°C [44]. This is followed by rapid cooling that protects the cells from extensive cell damage caused by slow cooling [50].

(i) Sucrose is a naturally occurring carbohydrates. Sucrose at negative temperature (−45°C) support preserved cells by required nutrition, and sucrose in combination with DMSO maintains good cytoprotective properties [48].

(ii) Trehalose (also called mycose or tremalose) is a natural non-reducing disaccharide consists of two molecules of glucose. Trehalose is less water soluble than sucrose, except at high temperatures (>80°C) and because of its high water-retaining capability, it can be used as cryoprotectant. The anhydrous forms of trehalose quickly reclaim moisture to form the dihydrate. Compared to standard freezing procedures, trehalose can improve cell vitality after thawing [49]. Disaccharides cryoprotectants are the best choice for EV-based therapeutics due to its safety and can be used for a wide range of proteins and cell products [41,51]. Using trehalose as cryoprotectant prevent formation of internal ice in biological particles, this led to prevention of EVs aggregation [52] and to increase their colloidal stability [53].

#### 6.1.4. Storage temperature and its effect on EVs viability

When incubated exosomes at −70°C were compare to similar exosomes stored at room temperature for 10 days, the exosomal protein, RNA and exosome marker were most reduced at room temperature compared with −70 and 4°C. Flow cytometry result showed that exosome population became more dispersed after room temperature incubation for 10 days compared with −70°C incubated or freshly isolated exosomes [54]. Studies recommended that temperature higher than −20°C not appropriate for intact exosomes preservation. Some results showed that the stability of different cell types including MSCs, EVs isolated from plasma and exosomal miRNA did not affected by repeated freezing and thawing [55]. Others stated that EVs structure could be liable as a result of phosphatidylserine repeated freezing and thawing [56].

### 6.2. Drying method preservation

*Preservation by dehydration of sample*. This technique depends on removal of water content by two different drying methods: (i) freeze drying and (ii) spray drying.

#### 6.2.1. Freeze drying

Freeze drying (lyophilization) is a two-step preservation method based on water freezing, followed by its removal. In another word, initially by sublimation (primary drying) followed by desorption (secondary drying) [57]. Sublimation is the basic principle involved in the freeze drying preservation process, where water basically passes from the solid state (ice) to the vapour state promptly without passing through the liquid state. This takes place under pressure (4.579 mm of Hg) and at temperature below 0.0099°C [58].

This process of drying is appropriate for substances that are stable in the dry state [59]. Lyophilization is the most suitable method to preserve thermo-liable materials as EVs, vaccines, viruses, proteins, peptides and colloidal carriers [60]. Freeze-dried material can be easily kept, in a constantly storable state and can be simply reconstituted by adding water [61].

##### 6.2.1.1. Principle of freeze drying

**Figure 1.**
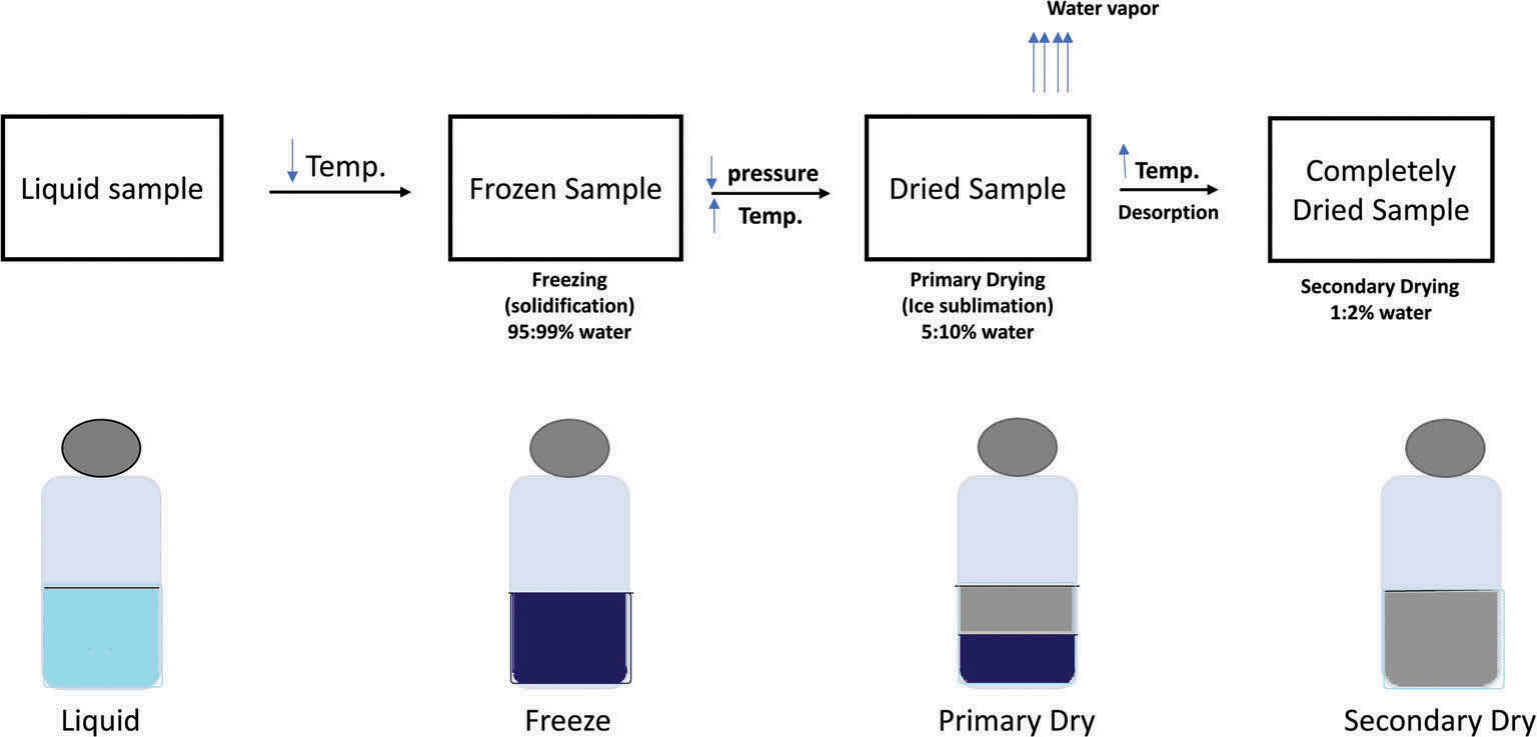
Schematic diagram of freeze drying

The main principle of freeze drying is sublimation, sublimation mean that water directly passes from solid state (ice) to the vapour state without passing through the liquid state. Lyophilization cycle starts with preparation of sample, freezing, primary drying (ice sublimation), secondary drying (desorption) and finally getting the lyophilized product [58] (Figure 1). The first step is freezing as frozen sample is necessary for low temperature drying. Vacuum is an important stage for sublimation, in which the frozen sample is placed under vacuum that allow solid water (ice) to become vapour (gas) without passing on liquid phase. Heat is required for accelerating sublimation. Then condensation, the low temperature condenser converts vaporized solvent which is obtained from the vacuum chamber to solid [62]. The final product is characterized with large surface area that promotes fast dissolution of the lyophilized product [63].

##### 6.2.1.2. Challenges associated with EVs lyo-preservation

Similar to cryopreservation, freezing and dehydration stresses generated during this process may lead to destructive effects on the biomolecules structure within the EVs, and thus mandates the use of a “lyoprotectant” in the formulation to protect the EVs and their cargo [56].

##### 6.2.1.3. Lyoprotectants

Lyoprotectant compounds occur in different formulations and aid different purposes. The lyophilized formulation is ruled by the guidelines determined by the active pharmaceutical ingredient (API) and the intended method of administration. These lyo-formulations may have one or more substances called “excipients” which accomplish one or more purposes. Excipients can be buffers/pH adjusters, bulking materials, stabilizing agents, or tonicity modifiers [64].

Buffers and pH adjusters are important to maintain the pH. The buffer choice is critical in the structuring of lyophilized preparations. For example, during freezing, phosphate buffers undergo severe pH changes. Using low buffer concentrations that undergoes minimal pH change during freezing such as citrate and histidine buffers can overcome severe pH deviations [58]. Bulking agents increase bulk to the lyo-formulation. They are used where very low concentrations of the active ingredient. These agents are often suited for small chemical drugs and some peptides but not efficient in stabilizing other products as emulsions, proteins and liposomes. Mannitol can be used in case of crystalline phase. Sucrose or other disaccharides can be used for proteins or liposomal products [58]. Disaccharides are the most common stabilizers used in lyophilization. They act by replacing the hydration sphere around the EVs by hydrogen bonding which interacts with phospholipid head groups and forms an amorphous sugar glass preventing fusion of products or protein destabilization [65]. Trehalose is listed as the most effective disaccharide to preserve EVs during lyophilization [52]. Mannitol-glycine, sucrose, sodium chloride and glycerol considered good tonicity modifiers which can be used in different situations to get an isotonic preparation that may be inclined by either the stability needs of the bulk solution or those for the method of administration [58].

The advantages of lyophilization include minimal chemical decomposition, product is stable at dry state and suitable for sterile operations. Disadvantages include prolonged time for handling and processing, unsuitable for volatile compounds and a sterile diluent is required for reconstitution.

#### 6.2.2. Spry drying

Spray drying is a suitable method to produce a variety of therapeutic agents such as vaccines, proteins and peptides for inhaled delivery [66]. Spray drying is initiated by atomizing the EVs solution, then by exposing to a heated gas, droplets are rapidly converted into a dry powder. If compared to freeze drying, spray drying is a faster, single step and drying method is designed as a continuous drying process so it’s more economical [67]. Spray drying is preferred for heat-sensitive constituents [67]. The dried product can be presented in different forms as powder, granules based on the dryer design as well as the physical and chemical properties of the substance [68].

Spray drying process consists of five steps: (1) Concentration: where the substance is normally concentrated before introduction into the spray dryer. (2) Atomization: according to the desired characteristics of the dried product the atomization stage creates the optimum condition for evaporation. (3) Droplet–air contact: in the chamber, atomized liquid gets in contact with a hot gas, leading to the evaporation of 95% of the water contained in the droplets within few seconds. (4) Droplet drying: the moisture evaporation occurs in two stages: *during the first stage*, there is enough moisture in the drop to replace the evaporated liquid at the surface and evaporation happens at a moderately constant rate; *the second stage* takes place when there is no longer enough moisture to maintain the saturated status at the droplet surface causing formation of dried shell around the surface of the droplet. (5) Separation: this is the final stage where cyclones, bag filters, and/or electrostatic precipitators are used.

##### 6.2.2.1. Applications of spray drying in pharmaceutical field

Many spray drying operations give a spherical particle, but others give non-spherical particles. Particles may be hollow or solid. Pressure spray nozzles can produce particles in size from 20 to 600 microns. Two-fluid nozzles generally produce particles in sizes from 10 to 200 microns and larger [69]. The forms of the final product can be: (1) *Granulation* characterized by better distribution of drug, improved flow colours and requires less lubricant than wet massed products. Spray drying gives a shell of concentrated binder at the surface of the granular material, make strong tablets and maximum use of binder [68]; (2) *Encapsulation* where the product is recovered about 15°C below the outlet temperature [70]. This suitable for microencapsulation of products such as antibiotics, vaccines, peptides and proteins; (3) *Inhalation* where highly specialized spray drying nozzles facilitate increased particle engineering capabilities, even in large scale making it possible to accurately manipulate the aerodynamic particle size and properties. Spray drying technologies make it easier than ever to efficiently produce therapies in the form of free-flowing particles that are ideally for inhalation [71]. Control release products creating a shell-like structure around the granular that allow spray drying to be used for the manufacture of controlled-release products.

In case of extracellular the atomization pressure and outlet temperature, can affect the stability of the EVs and their cargo. That considered critical process parameters must be identified and maintained within a narrow window [72].

## 7. Future perspectives of EVs

Given that EVs retain the properties of their originating cells, this requires standardizing stem cell culture conditions, EV isolation, identification, scaffold functionalization, and establishing the therapeutic benefit of this combination. To develop EV-mediated therapeutic, efficient, scalable bioengineering solutions are required; a progress is being made, but there remain technical challenges. Regardless of whether EVs will be used for the purposes of regenerative medicine, cancer vaccination, veterinary or agriculture, there is an obvious need to develop methods to reliably store, transport and apply the EVs.

## 8. Conclusion

Therapeutic EVs are considered a promising cell-free therapy, which can overcome the problems associated with cell therapy. The problem with fresh EVs solutions is that they can’t be kept for long periods of time and requires lengthy preparation time. Therefore, it was appropriate to investigate a preservation technique to preserve and extend the biological effect of these vesicles and to facilitate their clinical and commercial application. Preservation of EVs will initiate a vast amount of clinical trials on different species and different clinical problems. Although cryopreservation technique is characterized by simplicity and availability, some researches remarked that temperature above −20°C is not suitable for EVs preservation and repeated freeze and thaw may affect EVs. Dehydration appears to be a suitable technique for preservation. Freeze-dried EVs can be kept at room temperature, easily transported and prepared for clinical application, yet it requires more research to make the preparation fit for various administration routes. Spry drying is considered easy and cheap process but necessitates examining the effect of the heated gas used in the process on the EVs viability.
